# Legacy and live effects of ecosystem engineer differ across energy gradient, reversing net engineering outcome

**DOI:** 10.1007/s00442-026-05921-6

**Published:** 2026-08-28

**Authors:** R. Daniel Harris, James E. Byers

**Affiliations:** University of Georgia, 140 E. Green St, Athens, 30602 GA USA

**Keywords:** Estuaries, Facilitation, Foundation species, Physical-biological coupling

## Abstract

Ecosystem engineers influence abiotic and biotic properties of communities. However, environmental factors like stress or energy gradients could modify their influence. Furthermore, engineering effects include legacies that persist after the engineer’s death that may interact with gradients differently than the live engineer. Here, we show that live and legacy effects of the oyster *Crassostrea virginica*, a reef-building ecosystem engineer, act in opposing directions across a physical energy gradient, reversing oysters’ net engineering effect. When energy is low, live oysters primarily buffer erosional forces and increase habitat availability for cordgrass (*Spartina alterniflora)*. When energy is high, legacy effects of oysters on cordgrass are negative, because high energy piles dead oyster shells onto cordgrass and smothers it. Thus, with increasing energy, net effects of engineering on cordgrass shift from positive live effects to negative legacy effects. Estuary wide, oysters have a net positive effect on cordgrass, because high energy coastlines – where negative effects predominate – are rare. Live and legacy engineering can have disparate, context-dependent effects; combined, they determine the overall effects of engineers on communities.

**Supplementary Information:**

The online version contains supplementary material available at https://doi.org/10.1007/s00442-026-05921-6.

## Introduction

Biological systems are heavily influenced by physical abiotic processes. Although all species interact with physical variables, ecosystem engineers (hereafter: “engineers”; Jones et al. 1994, 1997) are particularly adept at modifying the physical processes in their environments, which in turn influence the engineers and other resident species. By structurally altering their physical environment, engineers influence co-resident species (Byers et al. 2006). For example, beavers (*Castor canadensis*), construct dams that restrict water flow, which ultimately creates a wetland upstream of the dam, resulting in physiochemical changes to the ecosystem (Wright et al. 2002). The physicochemical changes then drive changes in the community that inhabits that locality. Engineering can have both positive and negative effects on other species (e.g. Jones et al. 1997, 2010; Crain and Bertness 2006); however, the magnitude of these effects is typically a function of the physical intensity of the abiotic environment, which will determine the scope for abiotic modification that can occur (Byers 2024). For example, in systems with low physical input (kinetic energy), engineers have low engineering potential to modulate the physical environment; whereas, in systems with high input, engineering potential is greater (Crain and Bertness 2006; Jones et al. 2010).

Complementarity in species reactions to the environmental modulation of the engineer can mean an engineer affects one species positively while affecting another negatively. For example, a reef-forming coral can create habitat for species that thrive on hard substrata while reducing habitat for sediment-dwelling species. Further variation in effects caused by engineers can stem from differing degrees of environmental modification by engineers across a physical gradient (Jones et al. 2010). For example, the effect of a beaver dam on aquatic habitat characteristics is a function of water flow and channel slope (Byers 2024). The efficacy of the engineering and its impact vary with the amount of physical modulation being performed (Crain and Bertness 2006). In cases of strong environmental gradients, the influence of engineering on a given species could theoretically switch between positive and negative from one end of the gradient to the other.

Engineers affect their communities when they are alive, but they can also affect them after death. The latter are referred to as legacy effects (Jones, 2010; Smith et al. 2018; Kopecky et al. 2024). For example, beaver dams persist many years after the beaver dies or abandons the dam, termite mounds persist for decades after the mound is no longer used (Hastings et al. 2007). Through live and legacy effects, an engineering species can simultaneously have different interactions within a community. Furthermore, the time scales of live and legacy engineering effects can be different, given that legacy structural changes generally persist longer than the life span of the engineer (Hastings et al. 2007).

Legacy effects can also differ qualitatively from those that arise when the organism is alive. *Sabellaria alveolata* is a colonial polychaete tube worm that builds reefs in shallow coastal systems of western Europe. When alive, *S. alveolata* actively prevents epifauna and infauna recruitment to the reefs. When *S. alveolata* dies, reef biodiversity dramatically increases as local invertebrates colonize the now-unprotected reef structure (Firth et al. 2021). Differences between live and legacy effects can also occur when live and legacy structures interact differently with abiotic processes (Smith et al. 2018). For example, the extensive stationary vegetative stands often formed by estuarine marsh grasses attenuate waves and water currents, promoting marsh accretion. After the grass dies, however, the remnant floating rafts of dead grass are mobile and can smother live marsh plants (Smith et al. 2018). Such observed differences between live and legacy effects are not surprising, given that live engineers can dynamically respond to physical forces (e.g., by continuing to grow or maintain structure), while legacy engineering is inert (e.g., without active maintenance and subject to decay from physical forces).

Estuarine saltmarshes present ideal conditions to investigate multifaceted interactions between ecosystem engineers. These systems exhibit strong physical gradients in flow, temperature, inundation time, and salinity (Byers et al. 2015). As a result, these gradients set the stage for potentially large variation in engineering responses to changes along these physical gradients. Many marshes in the US Atlantic and Gulf coasts include two key species: *Crassostrea virginica* (hereafter: oysters), an ecosystem engineer and *Spartina alterniflora* (hereafter: cordgrass). Live oyster reefs have been shown to stabilize sediments and reduce shoreline erosion through wave and current (energy) dissipation (Bahr and Lanier 1981; Meyer et al. 1997; Grabowski et al. 2012), leading to protection and expansion of the cordgrass (Harris et al. 2023). However, after oysters die, their shells can persist in the system many years (Thompson et al. 2020), and when transported by currents, can accumulate into large shell rakes (deposited intertidal mounds of shell). Shell rakes are most common at higher elevation on estuarine banks exposed to high wave energy. These rakes engineer the environment as well, but their effects likely differ from those caused by living reefs because of their ability to be transported by energy, including waves and currents. For example, shell can be deposited on top of cordgrass stands, blocking light and water flow, and increasing cordgrass mortality (Crawford 2018) and spatial extent.

In this study we quantify the magnitude and net outcome of the coupled live and legacy effects of oysters on cordgrass habitat across a hydrological energy gradient. A strength of this study is that we examine these processes with detailed measurements that are conducted over the scale of an entire estuary with large enough physical forces to be visualized from aerial photography. Previous studies have suggested that cordgrass benefits more from protection provided by the oysters in high energy environments (Meyer et al. 1997; Piazza et al. 2005; Walles et al. 2015; Harris et al. 2023); however, shell rakes that accumulate primarily in high energy areas can smother and kill cordgrass (Bahr and Lanier 1981). Therefore, we hypothesize that across the increasing estuarine energy gradient, (a) the beneficial effect of live oysters on cordgrass will increase, while (b) the detrimental effect of legacy oyster shell will increase as the dead shell is aggregated in the marsh in greater quantity, and (c) these two opposing effects (of live and legacy oysters) will partly offset one another (i.e., partly cancel one another out). Therefore, we predict that the net effect on cordgrass will depend on the quantitative influence of these two distinct engineering aspects of oysters across the environmental energy gradient.

## Methods

To examine how the effect of oysters on cordgrass changes along a wave energy gradient, we utilized a suite of field surveys, remote sensing, and GIS analytic tools. We conducted this study in Liberty County, Georgia, west of St Catherines Island, between St Catherines Sound to the north and Sapelo Sound to the south (hereafter: St Catherines Estuary; 64km^2^ study domain; 31.660^o^ N; 81.235^o^ W). The study domain transitions from sounds on the Atlantic Ocean side, to brackish tidal creeks and tidal fresh rivers on the mainland side. This domain was chosen due to the existence of a near-complete inventory of oyster reefs in the domain, which was conducted in 2010 by the University of Georgia Marine Extension Service (MAREX, *N* = 802, Corley and Harris 2011; Power et al. 2010). The inventory mapped (via handheld GPS units) the perimeter of all live oyster reefs 1m^2^ or larger, provided that the site was accessible by boat at low tides (water channels > 13 m in width). Reefs separated by 1 m or more, were classified as separate reefs.

**Fig. 1:**
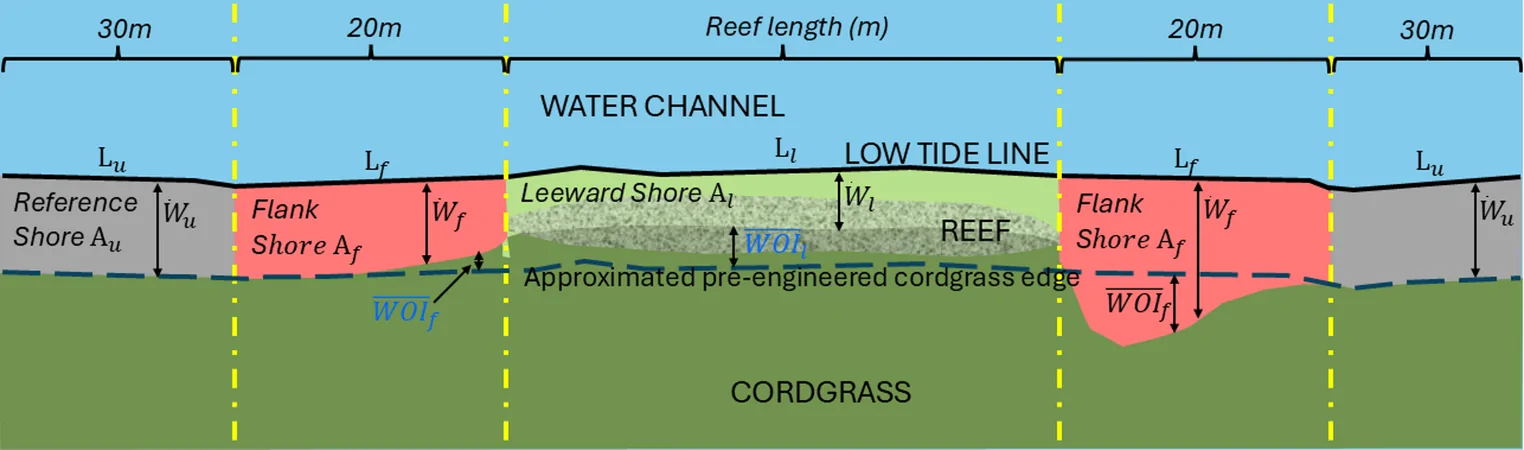
Aerial diagram of oyster (*Crassostrea virginica*) engineering effects on cordgrass (*Spartina alterniflora*) habitat. Areas (A) of non cordgrass intertidal habitats (i.e., oyster reefs and bare mud flats) are shown for un-engineered reference shore (grey, 50 to 20 m from reef edge, $\:{A}_{u}$), flank shore (pink, 20 to 0 m from reef edge, $\:{A}_{f}$) and leeward shore (light green, reef length, $\:{A}_{l}$), with the shore subsections separated by yellow dashed lines. Cordgrass (green), oyster reef (mottled grey), cordgrass/oyster overlap (mottled green), and water habitat (blue) are shown. Water’s edge at low tide (black line, L) is segmented according to the shore subsection it fronts: un-engineered, flank, and leeward (subscripts * u*, * f*, $\:\:l$ respectively). The average bank width ($\:\overline {WOI}$) for each subsection was calculated by dividing subsection area (A) by its length (L). The approximated location of the pre-engineered cordgrass habitat edge (blue dashed line) is shown. Mean leeward width of influence ($\:\overline {WOI}$_*l*_*)* is typically positive (blue); mean flank width of influence ($\:\overline {WOI}$_*f*_) can be positive (blue) or negative (black)

The study was conducted in two sections. The first section examines the mechanisms behind oyster engineering effect on cordgrass habitat width (i.e., perpendicular to the shore). Width is the dimension most influenced by interactions with the water and is also readily measurable. A small-scale study conducted by Harris et al. (2023), found that oyster engineering increases cordgrass habitat width by approximately 5 m in intermediate estuarine creeks (150–820 m wide) in Georgia. This study examines this relationship as a function of wave energy across the entire estuary. Cordgrass habitat edge is protected on the leeward (relative to hydraulic energy) side of oyster reefs (hereafter: leeward engineering; Harris et al. 2023). However, hardened structure, like oyster reefs, can create localized erosive eddies on the flanking ends of structures (hereafter: flank engineering). To take this into account we classified engineered shorelines as either flank or leeward engineered (Fig. 1). We record oyster engineering effects on cordgrass habitat directly behind (i.e., leeward of) the oyster reef patch (see Harris et al. 2023), and engineering effects that may occur on either side of the reef (i.e., flanking), beyond the reef patch. The second section builds off section one, by calculating at larger scale the total area of cordgrass habitat affected by live oyster reefs, dead oyster shell rakes, and the net effect of both for 1 km sections of coastline across an estuarine hydraulic energy gradient.

### The effect of live oysters on cordgrass habitat width as a function of wave energy

Live oyster reefs often extend farther out into the intertidal mudflats and tidal channels than do the surrounding bare banks (Harris et al. 2023; Fig. 1). The space immediately behind oyster reefs at higher elevation is usually filled with cordgrass (Bahr and Lanier 1981; Harris et al. 2023). We sought to measure this generally positive effect of oysters to increase cordgrass habitat width both behind and adjacent to reefs and quantify how it varied as a function of wave energy. We used six interconnected steps described in the following subsections to investigate this relationship:

*Wave energy*: As a proxy for wave energy, we quantified fetch (the uninterrupted distance over open water that wind blows in a single direction, allowing energy to transfer from the atmosphere to the water surface and generate waves) for all water in our study domain. First, we obtained 10 years (2006–2015) of daily wind data from a local weather station (https://forecast.weather.gov/data/obhistory/KSSI.html, see Keisling et al. 2020). Second, we created a water surface dataset for the study area from a digital elevation model (Alexander and Hladik 2015; 2 m pixel resolution). Lastly, we created a fetch dataset by quantifying weighted fetch (*F*, hereafter: fetch; Rohweder et al. 2012), for all water surface pixels as

1 $$\:F=\sum\:_{i=1}^{16}{w}_{i}\times\:{t}_{i}$$

where w_i_ is the length of water over which wind can blow without obstruction from the i^th^ direction, t_i_ is the fraction of the time that wind blows from the i^th^ direction (hereafter: wind direction time), and i refers to one of 16 cardinal wind directions (i = 0, 22.5, 45, … 337.5).

*Site selection*: We selected 30 sites to establish the relationship between fetch and oyster engineering effect on cordgrass habitat behind it. To do this we first extracted fetch data from our fetch dataset for the mid-point of all live oyster reefs in the dataset that were within our study domain (*N* = 802) using ArcGIS. Across all reefs, fetch ranged from 4 m to 1,986 m, with greater values indicating higher overall wind energy at the site. To select sites evenly across the fetch gradient, and thus yield a balanced (evenly weighted) analysis with a well-spaced independent variable, we subdivided this range into 30 bins, which resulted in a fetch range of 66 m for each bin, then randomly selected one reef from each bin for a total of 30 sites (Online Resource: Fig. S1).

*Coastline classification at each site*: Adjacent to each of our 30 reef sites we drew a 100-meter buffer around each reef (50 m on each side of each reef). Within this buffer, all coastline was either classified as un-engineered (50 m to 20 m from reef edge), flank engineered (20 m to 0 m from reef edge) or leeward engineered (length of the reef itself, Fig. 1). The 20 m distance used to designate flank shoreline was defined by measuring shore width as a metric of flank engineering from aerial photography (NOAA 2013) at 22 reefs (independent of our study sites). From that preliminary analysis we established that flank engineering within our study region does not extend beyond 20 m from reef edges (Online Resource text and Online Resource: Fig. S2). In fact, for the majority of sites the flanking effects are substantially less than 20 m. Our methodologies described in the following steps take this into account and do not over inflate these effects.

*Bank width*: We elected to measure the average bank width of non-cordgrass habitat (an inverse metric for cordgrass width) for each coastline subsection, because oyster and mud habitat signify the absence of cordgrass. Additionally, oyster and mud habitat are much easier to delineate as they are confined within a narrow lower intertidal shore band (Fig. 1). To measure non-cordgrass bank width for each classified coastline subsection at each site we used 4 steps in ArcGIS. (1) We digitized the bank region between the water’s edge and cordgrass edge within each site’s 100 m coastline buffer using aerial photography taken at low tide (NOAA 2013; Fig. 1). (2) We subdivided each site’s bank region into horizontal segments according to the three engineering classifications: un-engineered, leeward engineered, flank engineered (Fig. 1). (3) We calculated the shoreline length (L) and area (A) for each site subsection. (4) We divided the area of each subsection by its coastline length (A/L) to calculate the mean bank width (${\stackrel{-}{W}}$) for each subsection in meters. It is important to note that the NOAA (2013) imagery dataset is a mosaic of pictures taken as close to low tide as possible. Given that all subsequent GIS steps were relative measurements within sites, any differences in tidal exposure between sites were not a concern.

*Coastline reference assumption*: We compared cordgrass habitat under the influence of oyster engineering against un-engineered control sites that were beyond oyster reef influence (i.e., 20–50 m from reef, see “coastline classification at each site” section). Given that bank width at un-engineered shorelines in our study system is highly correlated with hydrologic energy (*p* < 0.0001, R^2^ = 0.56, Online Resource: Fig. S3), this baseline seems logical in that these areas are out of the sphere of influence of oyster reefs.

*Oyster reef width of influence*: To quantify the effects of leeward and flank engineering by oysters on cordgrass at each site, we estimated the change in cordgrass habitat width due to the presence of oyster reefs, or mean Width Of Influence ($\:\overline {WOI}$). $\:\overline {WOI}$ for leeward and flank cordgrass habitat was calculated by subtracting the mean leeward and flank bank widths from un-engineered bank width, using Eqs. 2 and 3, respectively,

2 $${\overline {WOI} _l}\left( {in\,\,{\rm{ }}meters} \right)\; = {{\bar W}_u}-{{\bar W}_l}$$

3 $${\overline {WOI} _f}\left( {in\,\,{\rm{ }}meters} \right)\; = {{\bar W}_u}-{{\bar W}_f}$$

where $\:\stackrel{-}{W}$ is average bank width, and subscripts * u*, * f*, and$\:\:l$ are engineering classifications (un-engineered, flank engineered, and leeward engineered, respectively, Fig. 1). Recognizing that bank width is the inverse of cordgrass width, positive $\:\overline {WOI}$ values indicate gains in cordgrass habitat and negative values indicate cordgrass habitat loss.

*Analysis*: To test if flank or leeward reef engineering was correlated to wave energy, we tested the effect of fetch (*F*) on flank ($\:\overline {WOI}$_*f*_), and leeward ($\:\overline {WOI}$_*l*_) reef engineering using linear regression ($\:\overline {WOI}$_*f*_ ~ *F*; $\:\overline {WOI}$_*l*_ ~ *F*, respectfully; *N* = 30). The initial models were not significant due to high variability in cordgrass habitat change at sites with high fetch (> 1,000 m, Online Resource: Fig. S4). Thus, we re-ran the models using sites with fetch values < 1,000 m that are far more abundant in the estuary. All analysis were conducted in R (R core team 2021; code/data: Harris 2024).

### The net effect of live oysters and shell rakes on cordgrass habitat area

Oyster reef and shell rake patch size and location, their engineering effects, and coastline fetch magnitude are not evenly distributed across the estuary. Thus, to quantify the net effect of oyster engineering on cordgrass habitat across an energy gradient, we calculated the live and legacy engineering effects of all oyster reefs and shell rakes across the entire study domain.

*Live engineering effects*: Given our large study domain and oyster reef dataset (*N* = 802) it was time prohibitive to directly measure the oyster engineering effects on cordgrass at each reef site. Instead, we used a two-pronged approach: (1) we estimated oyster engineering effects ($\:\overline {WOI}$_*f*_ and $\:\overline {WOI}$_*l*_ ) for all 467 sites with fetch values less than 1,000 m using the flank ($\:\overline {WOI}$_*f*_ ) and leeward ($\:\overline {WOI}$_*l*_ ) regression models developed in the previous section (which had good model fit with low variability, see Results section below). Fetch data for the midpoint of each reef (calculated in the previous section) was used to parameterize the regression models. The estimated $\:\overline {WOI}$_*l*_ engineering effects (in m) from the regression for each reef was then multiplied by the reef’s length (parallel to the shore) to estimate the area of leeward engineering effect (m^2^). The estimated $\:\overline {WOI}$_*f*_ engineering effects (in m) from the regression for each reef was multiplied by 40 m (20 m on each flank end of a reef) to estimate the area of flank engineering effect (m^2^). (2) Because the regression models performed poorly for reefs with fetch > 1,000 m, for the remaining 335 sites with fetch values over 1,000, we used aerial photography (NOAA 2013) to directly measure the area of oyster flank and leeward engineering effects (m^2^) on cordgrass.

**Fig. 2:**
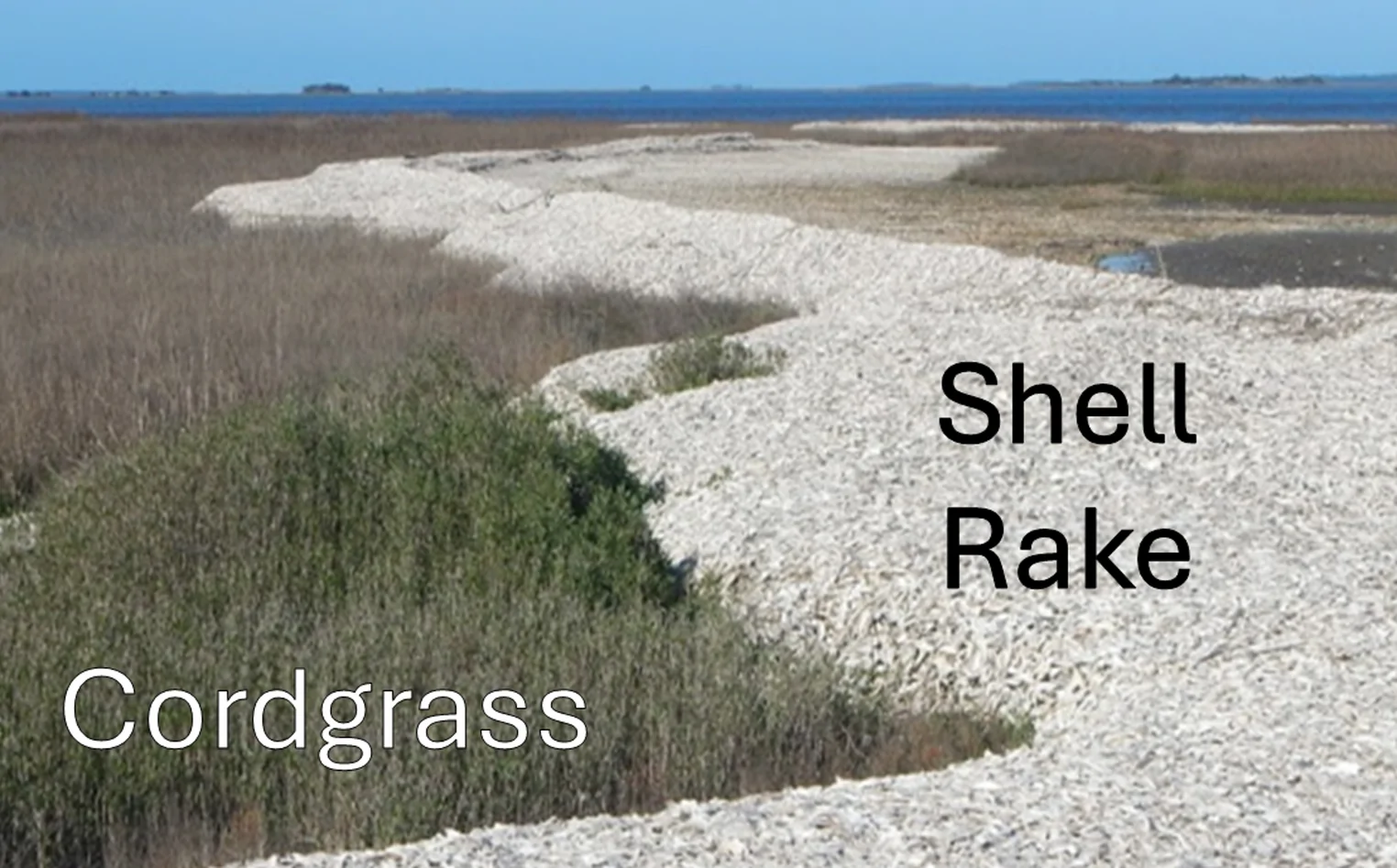
Side view (parallel to the waterline) of legacy oyster engineering. Dead oyster shell (rake) accumulates in deep deposits over thousands of years, and can be moved by hydraulic energy. When deposited over cordgrass habitat, live cordgrass patches are smothered and die

*Legacy engineering effects*: Given that cordgrass typically grows in monocultures of 100% cover, particularly near the lower elevational range of its habitat, here we assume that in the absence of a shell rake, we would find 100% cordgrass cover. Therefore, we assume that shell rake patch size area is equal to its (negative) engineering effect on cordgrass. To measure shell rake patch size, we used four band aerial photography (red, green, blue, and near infrared; NOAA 2013) to remotely map shell rakes. Due to the bleached white nature of dead shell (Fig. 2), we automated shell rake mapping by selecting “white” pixels (all pixels with a summed 4 band value > 800) between mean higher high water and mean sea level (typical cordgrass habitat elevation). For quality control, NOAA aerial photography was subsequently scanned manually for rakes and we detected there were no false negatives or positives in the automated process. Mapped shell rake edges were cleaned using ArcGIS tools (shrink-expand-shrink).

*Coastline segmentation*: Live engineering and legacy engineering sites do not occur at the same coastline elevations and are not evenly distributed across the study domain. To contrast the two forms of oyster engineering, we subdivided the coastline of the entire estuarine domain into 277 1 km segments (which included coastline elevations where live and/or legacy engineering, or neither takes place, Online Resource: Fig. S1 insert map). Within all segments we calculated the sum of all live, all legacy, and net (Σ(live) + Σ(legacy)) engineering effects (in m^2^). Fetch was sampled continuously (every 50 m) across each segment and the median fetch value for each 1 km coastline segment was recorded.

*Analysis*: To examine how live oyster reefs and shell rakes were distributed across the wave energy gradient, we modeled the probability of a 1 km coastline segment having oysters (presence/absence of oyster reefs and/or rakes) as a function of fetch with two GLM models (one each for live reefs and shell rakes) with binomial distributions. To build these models, we used all 277 segments, including segments without any form of engineering. McFadden pseudo R^2^ values were calculated for both models.

Next, to test how the effect of oyster engineering on cordgrass area changed across the wave energy gradient (i.e., area of cordgrass added or lost per 1 km coastline), we modeled the effect of fetch (*F*) on the area of cordgrass habitat partitioned into the effect of (1) live oyster reef (*N* = 277, Area_live_ ~ *F*), and (2) legacy shell rakes (*N* = 277, Area_legacy_ ~ *F*). We also looked at the total net effect by summing those components (*N* = 277, Area_live+legacy_ ~ *F*). We fit both linear and quadratic models to these three relationships with fetch. Thus, for all analyses we ran a second order polynomial and tested for fit and significance of the full model; if the polynomial term was not significant, it was dropped from the model and only the linear model was fit. Lastly, to summarize the collective amount of oyster engineering of cordgrass habitat that occurs across the entire estuary, we binned all 1 km coastline segments into three equal fetch categories (0–667 m, 667–1,333 m, 1,334–2,000 m). We used ANOVA to test if there were differences in net engineering between the three fetch categories. Pairwise treatment comparisons were tested using a Tukey post hoc test. All analysis was conducted in R (R core team, 2021; code/data: Harris 2024).

## Results

### The effect of live oysters on cordgrass habitat width as a function of wave energy

**Fig. 3:**
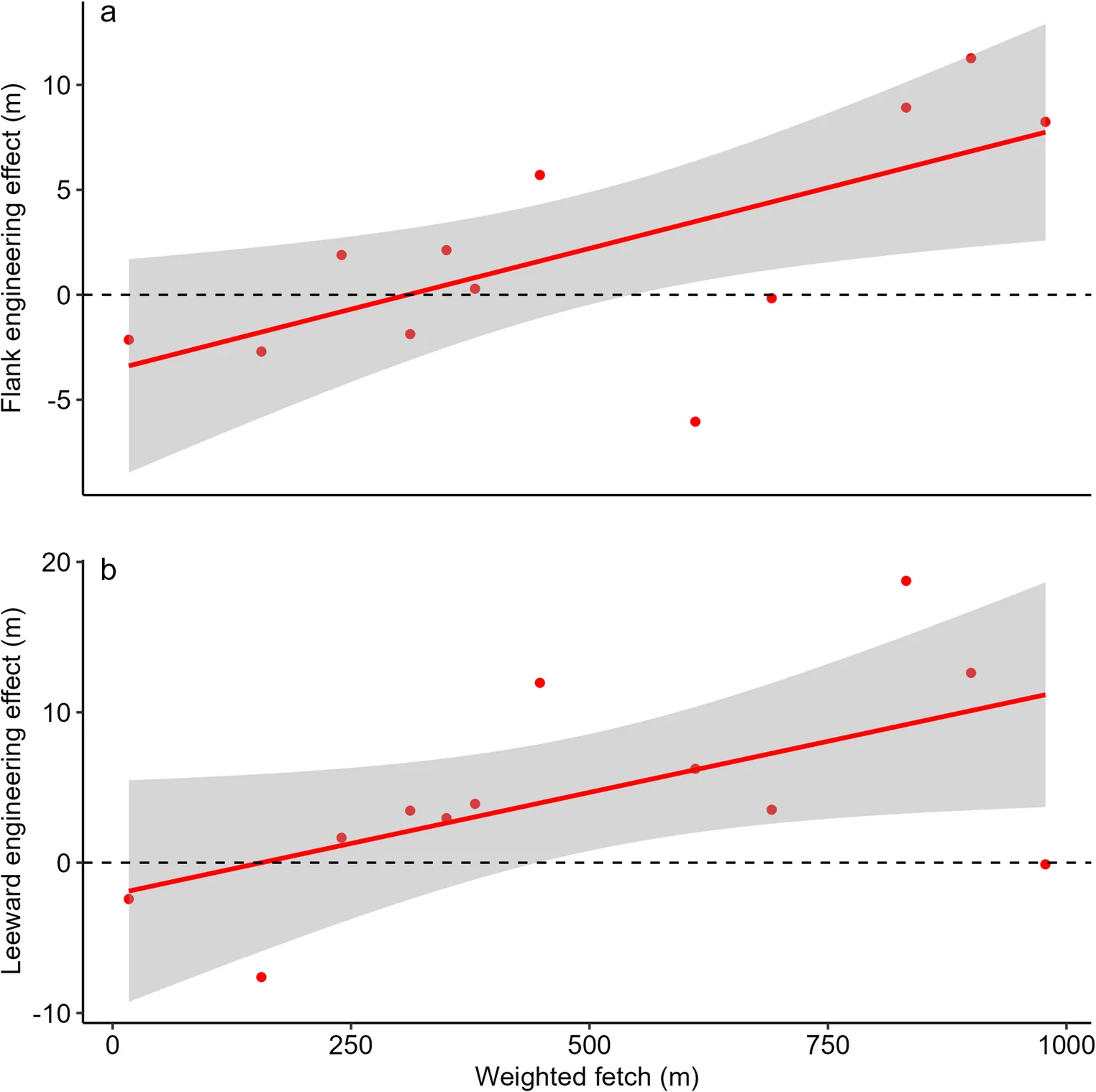
Effect of weighted fetch on oyster engineering of cordgrass habitat ($\:\overline {WOI}$). (**a**) Flank ($\:\overline {WOI}$_*f*_, F_1,10_ = 8.06, *P* = 0.02, r^2^ = 0.39) and (**b**) leeward ($\:\overline {WOI}$_*l*_, F_1,10_ = 5.28, *P* = 0.04, r^2^ = 0.28) engineering for sites with fetch values less than 1,000 m. Model regression lines (red), data points (red), and 95% confidence intervals (grey shading) are shown

Fetch did not significantly affect the flanking effects of oyster reefs on cordgrass habitat (F_1,28_ = 0.56, *P* = 0.46, r^2^ = -0.01, Online Resource: Fig. S4a), or the leeward effects of oyster reefs on cordgrass habitat (F_1,28_ = 0.17, *P* = 0.69, r^2^ = -0.03, Online Resource: Fig. S4b). Although these models were not significant, we did see a positive trend in both flanking and leeward effects for fetch values < 1,000 m. When the models were rerun using sites with fetch values less than 1000 m, the fit improved. Fetch (< 1,000 m) had a significant positive affect on oyster flank engineering (F_1,10_ = 8.06, *P* = 0.02, r^2^ = 0.39, Fig. 3a), and leeward engineering (F_1,10_ = 5.28, *P* = 0.04, r^2^ = 0.28, Fig. 3b), of cordgrass edge habitat. With increasing fetch up to 1,000 m, the effects of both flank and leeward oyster engineering on cordgrass habitat were positive, reaching a maximum $\:\overline {WOI}$ around 10 m (Fig. 3).

### The net effect of live oysters and shell rakes on cordgrass habitat area

**Fig. 4:**
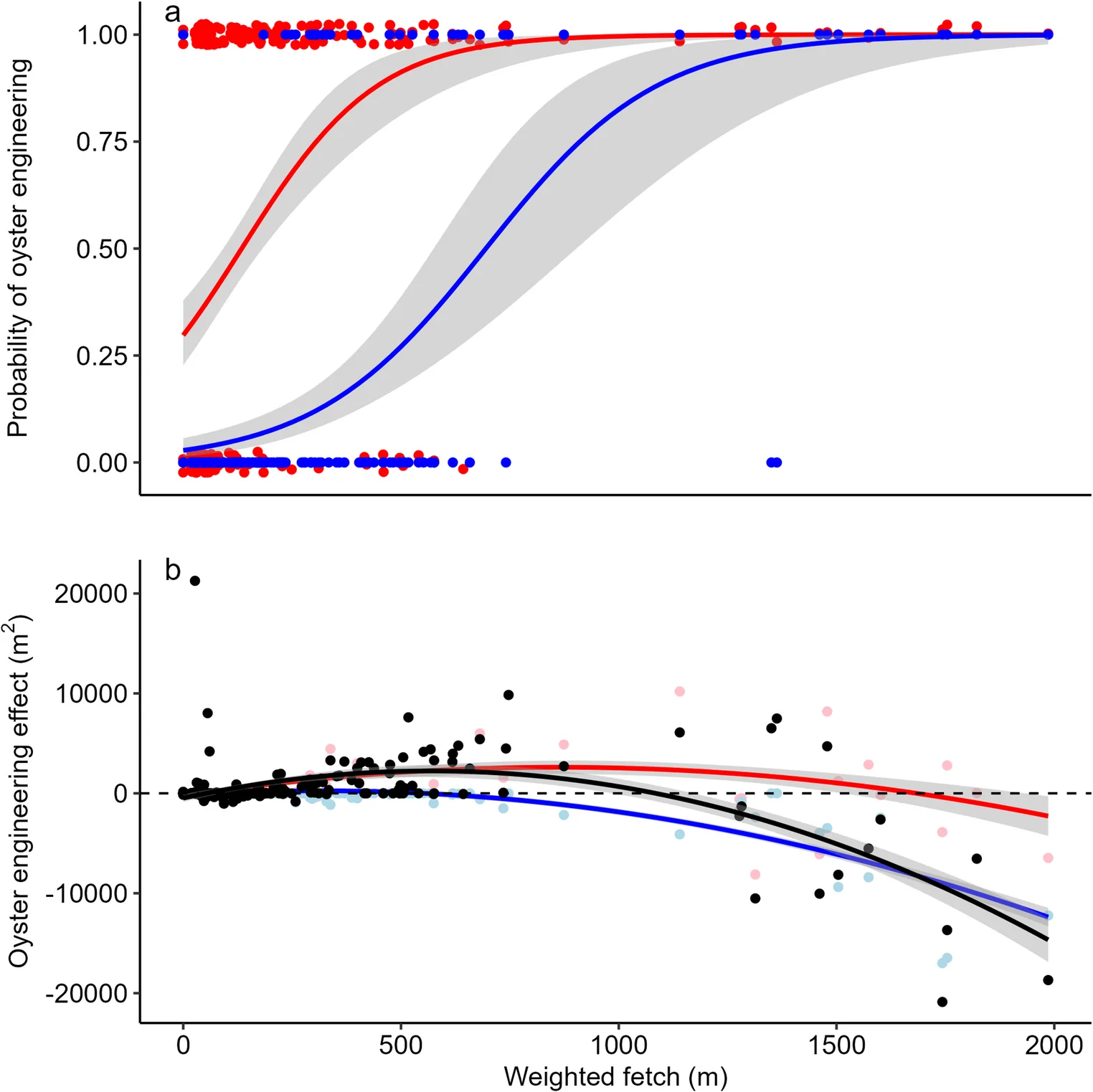
(**a**) Probability of cordgrass habitat engineered by live oyster reef (red, *P* = 0.0001, McFadden pseudo R^2^ = 0.18) and legacy dead shell rakes (blue, *P* = 0.0001, McFadden pseudo R^2^ = 0.39) as a function of fetch. Data points represent engineering presence/absence per 1 km segment of coastline. Model regression lines are shown in corresponding colors. (**b**) Magnitude of live (red regression lines and points, F_2,274_ = 22.66, *P* = 0.0001, R^2^ = 0.14), legacy (blue regression lines and points, F_2,274_ = 375.4, *P* = 0.0001, R^2^ = 0.73), and the net engineering effect on cordgrass area (black regression lines and black points, F_2,274_ = 89.7, *P* = 0.0001, R^2^ = 0.39) as a function of weighted fetch. Data points represent the summed area (m^2^) of live, legacy, and net (live + legacy) engineering of cordgrass area per 1 km segment of coastline

Across all 277 1 km shoreline segments, we enumerated 802 reefs and 182 rakes. Of all these 277 segments, 148 (53%) had live oyster reefs, 38 (14%) had oyster rakes, and 33 (12%) had both. Fetch had a significant positive effect on the probability of the occurrence of oyster reefs (*P* = 0.0001, McFadden pseudo R^2^ = 0.18, Fig. 4a) and the probability of occurrence of rakes (*P* = 0.0001, McFadden pseudo R^2^ = 0.39, Fig. 4a).

Both the linear and quadratic terms in the live reef, legacy (shell rake), and net engineering models were significant. Fetch (*F*) significantly affected the effects of oyster reefs on cordgrass habitat (F_2,274_ = 22.66, *P* = 0.0001, R^2^ = 0.14, effect of live oyster engineering on cordgrass area = *6*,*344 F – 12*,*384F*^*2*^ *+ 578*, Fig. 4b red). Fetch significantly affected the effects of legacy shell rakes on cordgrass habitat (F_2,274_ = 375.4, *P* = 0.0001, R^2^ = 0.73, effect of shell rake engineering on cordgrass area = *21*,*975 F + 14*,*521F*^*2*^ *+ 362*, Fig. 4b blue). Fetch also significantly affected the net effect of oyster engineering of cordgrass habitat (F_2,274_ = 89.7, *P* = 0.0001, R^2^ = 0.39, Net oyster engineering effect on cordgrass area = *-15*,*631 F – 26*,*905F*^*2*^ *+ 217*, Fig. 4b black). As fetch increased, net engineering switched from positive to negative at ~ 1,000 m.

**Fig. 5:**
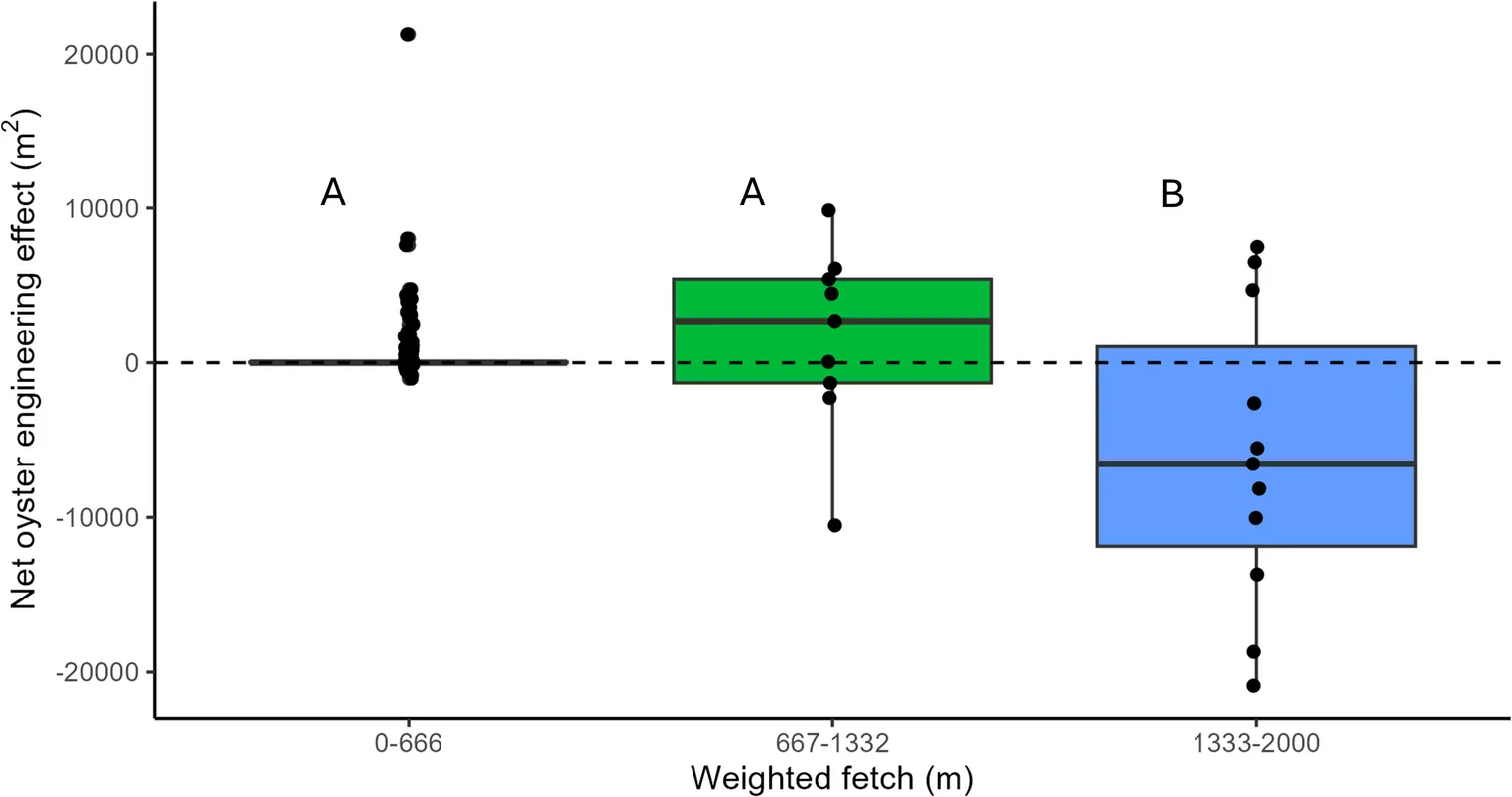
Net oyster engineering of cordgrass habitat at low (0–666 m, *N* = 257), mid (667-1,332 m, *N* = 9), and high (1,333-2,000 m, *N* = 11) fetch shorelines. Letters indicate significant treatment differences obtained from Tukey post hoc tests. Medians are depicted by the horizontal lines, plot-level data are depicted by the black dots, and the whiskers extend to 1.5 x the interquartile range

When summed over the whole study area (277 km of shoreline within a 64km^2^ study area), oysters facilitated the development of 59,993m^2^ of cordgrass habitat (0.22m^2^ net effect for every 1 m of shoreline), although the roles of the living and legacy effects were in opposite directions. Legacy effects (via shell rakes) removed 100,250m^2^ of cordgrass habitat, while living reefs had a net effect of creating 160,243m^2^ of cordgrass habitat. The majority of this positive engineering of cordgrass habitat arose in segments of the estuary with relatively low fetch (< 1,000), which were the most common (Fig. 4). Although the magnitude of engineering in any of these segments is small (Fig. 4), the large number of them boosts their collective engineering total. Areas with large fetch (> 1,000 m) are not common, but where they occur, net engineering decreases and changes sign, indicating a net loss of cordgrass habitat in those areas (Fig. 5).

## Discussion

In our survey of all the oyster reefs and rakes within a 64km^2^ study region we found a positive cumulative net effect of both live and legacy engineering on cordgrass habitat (a net increase of 59,993m^2^), with 28% of 1 km coastal segments demonstrating beneficial effects of oysters on cordgrass. Thus, the balance of oyster reefs and rakes had an overall positive impact upon the cordgrass footprint in the study area. At the same time, live and legacy engineering from reefs and rakes had different quantitative effects on cordgrass. Live engineering had a positive effect that initially increased as physical energy increased, but this positive effect decreased in magnitude at sites with high fetch (> 1,000 m). Because fetches < 1,000 m predominate in the estuary, net positive effects of oysters on cordgrass are most common. Thus, overall, live reefs primarily affected cordgrass footprint positively, while legacy effects of shell rakes affected it negatively. In the few coastal segments with high wave energy, live effects of oyster reefs on cordgrass became negative, and the magnitude of negative legacy engineering from shell rakes was high, changing the net oyster engineering effects on cordgrass habitat to be substantially negative (Fig. 4b).

Oysters, as autogenic ecosystem engineers, create structural habitat through the very nature of their presence (for example, by affecting the habitats of oyster-associated organisms like crabs and mussels, e.g., Byers et al. 2017; Grabowski et al. 2020; Zu Ermgassen et al., 2020), and through the extended engineering effect that their presence exerts on other habitat nearby (for example, by expanding cordgrass habitat in the marsh). A previous study (Harris et al. 2023), found that oyster reefs in intermediate estuarine creeks (150–820 m wide) were associated with slightly wider cordgrass habitat. In this study we have strengthened and quantified that association, finding that positive effects between oysters and cordgrass increase with increasing physical energy up to a point where fetch is ~ 1,000 m. We also found that oyster reef effects became negative for coastlines with high fetch (> 1,600 m). We believe this tipping point may be due to a qualitative change in the oyster response to the magnitude of physical energy in the system. Specifically, at low to moderate levels of energy (*F*), the positive relationship of oyster reef effects with fetch is congruent with ecosystem engineer theory, whereby the influence of engineering increases in more physically energetic environments, because there is more scope for the engineer to alter or ameliorate increasing physical stress (Crain and Bertness 2006; Byers 2024). However, this amelioration could be dampened or overpowered in high energy environments, where strong physical conditions may limit the engineers’ buffering capacity or even the engineers themselves. Nevertheless, at the estuary scale, because of the predominance of lower energy sites (Fig. 4), oysters have an overall net positive effect on cordgrass. The influence of oysters on cordgrass is essential to ascertain because collectively these two foundational species influence the majority of the species in this estuarine system.

Shell rakes, on the other hand, smother cordgrass, resulting in a decrease in cordgrass habitat equal to the patch size of the shell rake. We found a strong positive relationship between shell rake size and wave energy (F_2,274_ = 375.4, *P* = 0.0001, R^2^ = 0.73). As hydraulic energy in the form of fetch increased, so did the size of shell rakes, because dead shell is mobile and increasingly likely to be deposited as large rakes in cordgrass habitat with high wave energy. Although the shell rake smothers cordgrass, it is still possible that shell rakes might benefit cordgrass nearby by dissipating wave energy. Although this consideration lies beyond the scope of our present study, we would suggest that such beneficial effect is likely to be minimal, given that the rakes normally occur at high tidal elevations where water volume and flow is normally low.

Engineering magnitude – that is, the degree to which oyster reefs and rakes influence cordgrass habitat – is also affected by the interaction between spatial scale and wave energy distribution within the system. We have shown that at the estuary scale there is a net positive effect of oyster engineering. This comes in spite of the fact that at the 1 km scale (Fig. 4), net engineering as a function of fetch is unimodal, with negative engineering values at high fetch values. This difference is explained by the fact that most estuarine 1 km segments have low to intermediate fetch (Fig. 5), and thus why the positive effects of engineering dominate at the landscape scale.

Our study demonstrates that live and legacy effects, of oyster reefs and rakes upon cordgrass, are not necessarily the same in degree or location. The contrast in effects of live and legacy engineering may stem from the fact that the oyster is an autogenic ecosystem engineer, meaning that when alive, its own body provides the physical structure that engineers the environment. Upon death, its dead body parts take on new properties such as orientation and position within the environment, setting up the possibility for new engineering effects. Most autogenic engineers are sessile for the duration of their life cycle, but their structures often become more mobile after death (Smith et al. 2018, 2021). As a case in point, oyster shell rake material is the result of estuary-wide accumulation of legacy shell over the course of millennia (Thompson et al. 2020), whose placement high in the intertidal zone and effect upon cordgrass habitat is not necessarily related to the present-day location of live reefs. As another example, aquatic vegetation is sessile and grows upright, but falls over after death or becomes detached from the substrate and is mobilized through water and wind. Sessile upright structures will likely interact with the physical environment differently than structures that are more mobile and horizontal. For comparison, because allogenic engineers modify or create structure external to their own bodies, the live and legacy engineering are typically one and the same, the only difference being that legacy engineered structures are no longer maintained. For example, beaver dams and termite mounds are structures that can persist with their form largely intact for many years after the engineer is extirpated.

Given these considerations, we hypothesize that observed differences between live and legacy engineering effects of autogenic engineers are likely to be most pronounced when the legacy effect is mobile, long lasting, and in a different micro-orientation from the live engineer. Reef-forming bivalves often exhibit these traits (Gutiérrez et al. 2003). As a result, we found different positioning and quantitative effects of live and legacy oyster engineering as a function of fetch. Live and dead oysters end up in different places, and this positioning of the oysters determines much of their engineering influence. Namely, live reefs would smother cordgrass if reefs were positioned higher in the marsh, and shell rakes would likely protect cordgrass edges if the rakes were positioned in the lower intertidal zone armoring the intertidal bank from erosion. However, these opposite positionings do not naturally occur because the oyster engineer in its live and dead stages interacts with the physics of the environment in different and specific ways.

Live and legacy engineering effects on species and communities may differ. In the oyster-cordgrass system when wave energy is low, live effects are positive, while legacy effects are negative and low in magnitude. However, when wave energy is highest, legacy effects are highly negative, and live effects become negative. Thus, the quantitative differences in the relationships of live and legacy effects with energy are highly influential, and result in a net engineering effect that switches sign across the energy gradient. We propose that trait differences between the live and dead engineer may help predict when their engineering effects will differ in important ways.

## Supplementary Information

Below is the link to the electronic supplementary material.

Supplementary Material 1

## Data Availability

The locations of all data and code used in this manuscript are reported in the manuscript (Harris 2024 Data/Code: Legacy and live effects of ecosystem engineer differ across energy gradient, reversing net engineering outcome. Zenodo. https://doi.org/10.5281/zenodo.14053201*).*
