# Supplementary material for: Legacy and live effects of ecosystem engineer differ across energy gradient, reversing net engineering outcome
Source: Oecologia. 2026 Aug 28;208(9):121. doi: 10.1007/s00442-026-05921-6 (PMC13524962; doi:10.1007/s00442-026-05921-6)
Supplement: Supplementary file 1 [file 442_2026_5921_MOESM1_ESM.docx]

**Supplemental Material Title Page**

**Journal name:** Oecelogia

**Manuscript type:** Original Research (Topic: Aquatic Ecology)

**Manuscript title:** Legacy and live effects of ecosystem engineer differ across energy gradient, reversing net engineering outcome

**Author names for publication:** R. Daniel Harris, James E. Byers

**Affiliation(s) for each author:** University of Georgia, 140 E. Green St., Athens, GA 30602, U.S.A.

**Corresponding author:** R. Daniel Harris

**Corresponding author email address:** robert.d.harris@gmail.com

**Open Research statement:** The locations of all data and code used in this manuscript are reported in the manuscript (Harris, R. D. (2024). Data/Code: Legacy and live effects of ecosystem engineer differ across energy gradient, reversing net engineering outcome. Zenodo. <https://doi.org/10.5281/zenodo.14053201>).

**Authors' contributions:** RDH and JEB conceived the ideas and designed methodology; RDH collected the data; RDH and JEB analyzed the data; RDH led the writing of the manuscript. All authors contributed critically to the drafts and gave final approval for publication.

**Key words/phrases:** Estuaries, facilitation, foundation species, physical-biological coupling

**Online Resource Text**

Intertidal hardened structures like manmade bulkhead shorelines or natural oyster reefs can affect shorelines downstream of the hardened structures footprint. This is known as flanking effects. To assess the linear shoreline extent of oyster flank engineering (either side of oyster reefs, also see Fig. 1) as a function of fetch, we selected 22 sites across a fetch gradient from St Catherines Estuary, Liberty County, Georgia, (64km^2^ study domain; 31.660^o^ N; 81.235^o^ W). We created a 100 meter buffer of shoreline around each reef. Cordgrass and water’s edge were digitized from NOAA (2013) aerial photography. Starting at the outer buffer limit and working inwards towards the reef, shore width (distance between cordgrass and water’s edge) was measured every 5 meters. When width increased by 2 meters or more, we measured shore width every meter, to more finely resolve the extent of flanking effects. The shoreline distance between the reef edge and outer limit of flanking effects was measured for each reef. We found that at all sites, flank effects were contained within a 20-meter buffer of oyster reefs (Appendix: Fig. S2). We also found that fetch was not correlated to the linear extent of flank engineering (F_1,20_ = 1.69, *p* < 0.21, R^2^ = 0.03). It is important to note, that while we found that flanking effects could occur up to 20 meters from a reef, most reefs did not have any flanking effects

**Online Resource:** **Fig. S1** Aereal view of study site around St Catherines Island, Ga. The 30 sites (green dots) used in “The effect of live oysters on cordgrass habitat width as a function of wave energy” section of the study are marked. Additionally, the 1km shore segments (green shaded areas in the bottom left insert map) used in “The net effect of live oysters and shell rakes on cordgrass habitat area” section of the study are shown

**Online Resource:** **Fig. S2** Linear (shore-parallel) extent of oyster flank engineering as a function of fetch. At all 22 sites, the linear extent of oyster flank engineering was under 20m and usually much less than 10m. Model regression lines (red), data points (red), and 95% confidence intervals (grey shading) are shown

**Online Resource:** **Fig. S3** Shore width at reference un-engineered shorelines as a function of fetch. Model regression lines (red), data points (red), and 95% confidence intervals (grey shading) are shown

**Online Resource:** **Fig. S4** Effect of weighted fetch on oyster engineering of cordgrass habitat ($\bar{WOI}$). (a) Flank ($\bar{WOI}$*_f_*) and (b) leeward ($\bar{WOI}$*_l_*) engineering for all 30 sites. Sites were chosen to represent all weighted fetch classes (and not based on the commonality of weighted fetch classes). Increased variability in engineering effects for sites with mid to high fetch, can be seen (a and b). Model regression lines (red), data points (red), and 95% confidence intervals (grey shading) are shown
